# Effect of Interface Transition Zone and Coarse Aggregate on Microscopic Diffusion Behavior of Chloride Ion

**DOI:** 10.3390/ma15124171

**Published:** 2022-06-12

**Authors:** Jing Liu, Xuandong Chen, Hua Rong, Aiping Yu, Yang Ming, Ke Li

**Affiliations:** 1 College of Civil and Architecture Engineering, Guilin University of Technology, Guilin 541004, China; 6614062@glut.edu.cn (J.L.); apyu@glut.edu.cn (A.Y.); 2019048@glut.edu.cn (Y.M.); likeniko@126.com (K.L.); 2 Guangxi Key Laboratory of New Energy and Building Energy Savin, Guilin University of Technology, Guilin 541004, China; 3 Collaborative Innovation Center for Exploration of Nonferrous Metal Deposits and Efficient Utilization of Resources, Guilin 541004, China; 4 Guangxi Engineering and Technology Center for Utilization of Industrial Waste Residue in Building Materials, Guilin 541004, China; 5 Central Research Institute of Building and Construction, Beijing 100082, China

**Keywords:** chloride ion attack, multiscale model, concrete durability, concrete meso-structure

## Abstract

Concrete is a multiphase composite material composed of coarse aggregate, cement mortar, and interface transition zone (ITZ). It is of great significance to study the effect of ITZ and coarse aggregate on chloride microscopic diffusion behavior for predicting the service life of reinforced concrete (RC) structures. By introducing the random distribution function, a random coarse aggregate model considering the randomness of the thickness of the ITZ was established. Furthermore, a two-dimensional (2D) chloride ion diffusion mesoscopic model was developed by specifying different diffusion properties for different phase materials of concrete. Moreover, the effects of coarse aggregate rate, ITZ thickness, and ITZ diffusion property on chloride ion diffusion behavior were investigated in this paper. The research showed that the aggregate has hindrance and agglomeration action on chloride ion diffusion. Although the volume content of the ITZ was very small, less than 0.2% of the total volume of concrete, the effect of the ITZ on the chloride diffusion in concrete cannot be ignored. More importantly, the mechanism of promoting chloride diffusion in the ITZ was revealed through the chloride diffusion trajectory. The research revealed the transmission mechanism of chloride ions in the meso-structure of concrete and provides theoretical support for the design of RC structures in coastal areas.

## 1. Introduction

Chloride ion attack is one of the main factors causing durability failure of marine structures [1]. It is of great significance to investigate the diffusion behavior of chloride ion in concrete. In recent decades, much theoretical [2,3], experimental [4,5,6], and numerical [7,8,9] research on chloride ion diffusion in concrete have been done, and remarkable research findings also have been achieved. For example, CollePardi et al. [10] established the theoretical basis model of chloride ion diffusion in concrete by introducing Fick’s Second Law. As well, Mangat et al. [10] derived a modified equation considering the change of diffusion coefficient with time based on CollePardi et al.’s work. Moreover, Fu et al. [10] adopted the immersion test to measure the chloride ion concentration distribution through drilling powder sampling. Yu et al. [11,12] have also established a numerical model based on theoretical equation and chloride diffusion coefficient measured by test and carried out parametric analysis. Moreover, the reactive transport modelling was developed by Guo et al. [13] to investigate chloride ingress in coral aggregate concrete. However, concrete is a multiphase material composed of aggregate, cement paste, ITZ and pores [14,15]. The comprehensive influence of each component on chloride ion diffusion forms the macroscopic physical phenomenon, which leads to the randomness of the chloride diffusion coefficient [16]. Therefore, it is particularly important to study the effects of different components on the diffusion properties of chloride ions from the meso-level.

The interfacial transition zone (ITZ) is a thin layer of the transition zone between aggregate and cement paste [17,18]. The formation of ITZ is the result of the wall effect of the cement particles accumulation along the aggregate surface and the hydration of cement particles. Hence, the larger porosity in ITZ is due to the fact the cement content in the region is relatively low, which leads to large porosity and good connectivity. Bourdette et al. [19] measured the pore structure distribution of ITZ by mercury injection test (MIP), and found that the porosity in the ITZ is 2–3 times higher than that in the cement paste. However, high porosity and connectivity provide convenient conditions for water and ion transport. Yang et al. [20,21] and Ye et al. [22] studied the influence of ITZ on chloride ion diffusion by using prefabricated cylinders instead of aggregate. The study showed that the chloride ion diffusion coefficient of ITZ was about 30–50 times that of cement paste, indicating that the existence of ITZ greatly promoted the chloride ion permeability. Moreover, Huang et al. [22] used a similar method to investigate the influence of ITZ on water transmission, and found that ITZ has high permeability. The permeability coefficient of ITZ is 20 times that of ordinary cement paste.

In addition, the thickness of the ITZ is another important factor for the chloride ion diffusion property of concrete. Wu et al. [23] measured the average thickness of the cylindrical-aggregate ITZ by electron microprobe technique and the average thickness of ITZ is about 40.5 μm. Furthermore, Scrivener et al. [23] measured the thickness of the ITZ by SEM technology and find that the thickness distribution of the ITZ is random, ranging from 30 μm to 100 μm, and it has the characteristics of random distribution. Kim et al. [24] also found that the thickness of the ITZ is between 50 μm and 100 μm by analyzing the BSE images. Due to the thickness of ITZ being random and uncontrollable, it is difficult to control the thickness of ITZ by experiment. At present, many scholars use the total volume and surface area of ITZ to characterize the effect of ITZ thickness on chloride diffusion performance. Yang et al. [20] measured the effective chloride diffusion coefficient of concrete by accelerated chloride migration test to study the relationship between the surface area of ITZ and chloride diffusion coefficient. As well, the research shows that with the increase in surface area of ITZ. Zheng et al. [25,26] adopted the concentric circle model and deduced that with the increase of the ITZ thickness, the effective diffusion coefficient of the concrete block also increased. Therefore, the ITZ has a very important influence on the chloride ion diffusion in coagulation.

At present, the influence of ITZ on chloride diffusion performance is only limited to using cylindrical aggregate to replace the real shape aggregate through experiments. Moreover, due to the limitations of the experiment, it is difficult to reveal the mechanism of the influence of ITZ on chloride transport performance by experimental method. A multi-phase mesoscopic numerical model is proposed to fully explore the mechanism of diffusion behavior of chloride in concrete for predicting the service life of RC structures. As well, the effects of coarse aggregate rate, ITZ thickness, and ITZ diffusion property on chloride ion diffusion behavior are investigated in this paper.

## 2. Model Establishment

### 2.1. Concrete Mesostructure

**Figure 1:**
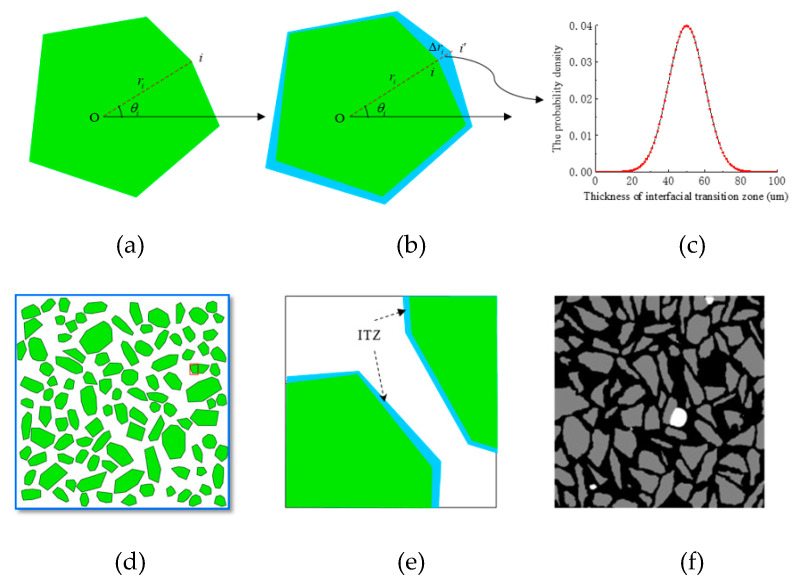
Concrete mesoscopic model generation. (**a**) A single aggregate, (**b**) Generate ITZ, (**c**) ITZ thickness probability distribution, (**d**) mesoscopic aggregate model, (**e**) Local magnification, (**f**) CT scanning of concrete [7], respectively.

In this paper, it is assumed that the thickness of the ITZ obeys a normal distribution ($X∼N\left(\mu ,\sigma^{2}\right)$), and based on the existing random aggregate model [27,28,29,30], a new concrete random aggregate algorithm model is established to consider the randomness of the ITZ thickness. The following steps describe the formation process of mesoscopic model in detail. First, the concrete random aggregate model of Gao et al. [31] is used to generate a single aggregate without ITZ and the aggregate area and the distance ($r_{i}$) from each vertex to the center are recorded, as shown in Figure 1a. It is worth noting that the details of the random generation method for individual aggregates can be found in reference [31]. Secondly each vertex is topologically $\Delta r_{i}$ outward along the radial direction, and $\Delta r_{i}$ obeys a random number of normal distribution ($X∼N\left(\mu ,\sigma^{2}\right)$), where μ is the average value of the ITZ thickness. Then connect the topological points in turn to form the boundary of the ITZ, as shown in Figure 1b. ITZ is also surrounded by the outer boundary of ITZ and outer boundary of aggregate. Thirdly, an aggregate library containing random ITZ thicknesses is generated, and the equivalent diameter of each aggregate is recorded by equal-area calculation [7]. Finally, according to the equivalent diameter and Fuller gradation cumulative distribution function [32], the aggregate was randomly selected from the aggregate library for delivery. And conduct interference inspection to finally form the target two-dimensional random aggregate, as shown in Figure 1d. the local magnification, and the distribution of the ITZ can be clearly seen as shown in Figure 1e. Figure 1f shows CT scan sections of concrete by Yu et al. [33]. By comparing Figure 1d,f, it can be found that the new concrete random aggregate algorithm model proposed in this paper is similar to the aggregate distribution of the actual CT scan section of concrete.

It is worth noting that the diameter of each polygon aggregate equivalent to a circular aggregate is determined. As well, the diameter of each polygon aggregate can be expressed as [30]:

(1) $$P\left(d\right)=1.065{\left(d/d_{m}\right)}^{1/2}-0.053{\left(d/d_{m}\right)}^{4}-{\left(d/d_{m}\right)}^{6}-0.0045{\left(d/d_{m}\right)}^{8}-0.0025{\left(d/d_{m}\right)}^{10}$$

where d is the diameter of aggregate; $d_{m}$ is the maximum diameter of aggregates; P is the cumulative percentage passing a sieve with aperture diameter d. In the revised manuscript, we also made the corresponding adjustment.

### 2.2. Multiscale Model of Chloride Diffusion 

**Figure 2:**
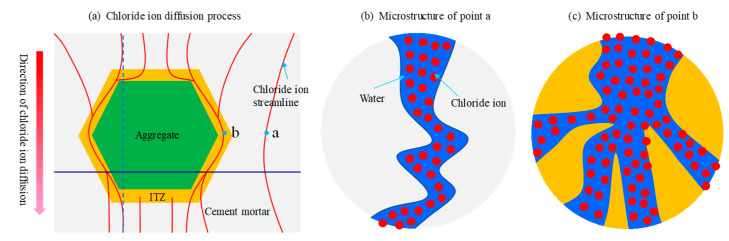
Chloride ion microscopic diffusion schematic diagram (**a**–**c**) respectively.

Generally, the microstructure of aggregate is very dense and the connectivity of pore structure is poor, comparing with chloride ion diffusion in cement mortar phase or ITZ, the chloride diffusion behavior in coarse aggregate phase can be ignored. Hence, this paper only considers the chloride diffusion behavior in the cement mortar region and the interface transition region. Figure 2 shows the microscopic diffusion process of chloride ions in representative elements. Under the action of concentration difference, chloride ion diffuses from the high concentration zone to the low concentration zone. The channel of chloride ion diffusion is the micro connectivity pore structure in concrete. The diffusion coefficient is related to the connectivity and tortuosity of pore structure. During the pouring of concrete, due to the vibration and the wall effect of aggregate, the pore structure in the ITZ has better connectivity and larger porosity than that in the cement mortar phase, which is conducive to the diffusion of chloride ions. The flow diagram of chloride ion diffusion is demonstrated in Figure 2. As mentioned above, this phenomenon is essentially caused by the difference in microscopic pore structure.

The pore microstructure diagram of cement mortar and ITZ are demonstrated in Figure 2b,c, respectively. Several scholars have done a lot of experimental research about chloride diffusion coefficient in cement mortar and ITZ. For details, please refer to literature [6,34,35], which is not cumbersome here. However, in the existing studies, the chloride diffusion coefficient is the average value at a macro level, which cannot be directly used for the study of microstructure. In fact, the pore microstructure of concrete is very complex and random [36], so it is difficult to describe the chloride diffusion in the pore structure with a certain value. In this paper, a random variable φ(x,y)  is introduced to characterize the randomness of pore size and connectivity between pores, which is mainly determined by the hydration process of cement paste. Thus, the chloride diffusion coefficient in meso-structure is obtained by multiple coupling methods, as shown in Equations (1) and (2) [37,38,39].

(2) $$D_{cem,cl}=\frac{2\times f_{p}^{2.75}D_{p}}{f_{p}^{1.75}\left(3-f_{p}\right)+n{\left(1-f_{p}\right)}^{2.75}}\times {\left(\frac{t_{\mathrm{ref}}}{t}\right)}^{m}\times \exp\left[\frac{U_{c}}{R}\left(\frac{1}{T_{ref}}-\frac{1}{T}\right)\right]{\left[1+\frac{{\left(1-h\right)}^{4}}{\left(1-h_{c}\right)}\right]}^{-1}\times \varphi_{cem}\left(x,y\right)$$

(3) $$D_{ITZ}=\;D_{ITZ0}\left(0.001+0.07\;\phi_{ITZ}^{2}+1.8\cdot H\left(\phi_{ITZ}-\phi_{cri}\right)\cdot {\left(\phi_{ITZ}-\phi_{cri}\right)}^{2}\right)\cdot \;\varphi_{ITZ}\left(x,y\right)$$

where $D2_{cem,cl}$ is diffusion coefficient of chloride ion in cement mortar; $D_{ITZ,cl}$ is diffusion coefficient of chloride ion in the ITZ; $U_{c}$ is the chloride ion diffusion activation energy, ($U_{c}$ = 44.6KJ/mol); *t* is the reference exposure time; *R* is the gas constant, (*R* = 8.314J/(K·mol)); $T_{ref}$ is the reference temperature; *h* is relative humidity; *h_c_* is critical humidity (*h_c_* = 0.75); $\phi_{ITZ\;}$ is the porosity of ITZ; *H* is the heaviside function; $\phi_{cri}$ is the critical porosity $\varphi_{cem}\left(x,y\right)$, $\varphi_{ITZ}\left(x,y\right)$ is a random number subject to normal distribution.

## 3. Numerical Simulation

### 3.1. Numerical Solution

**Figure 3:**
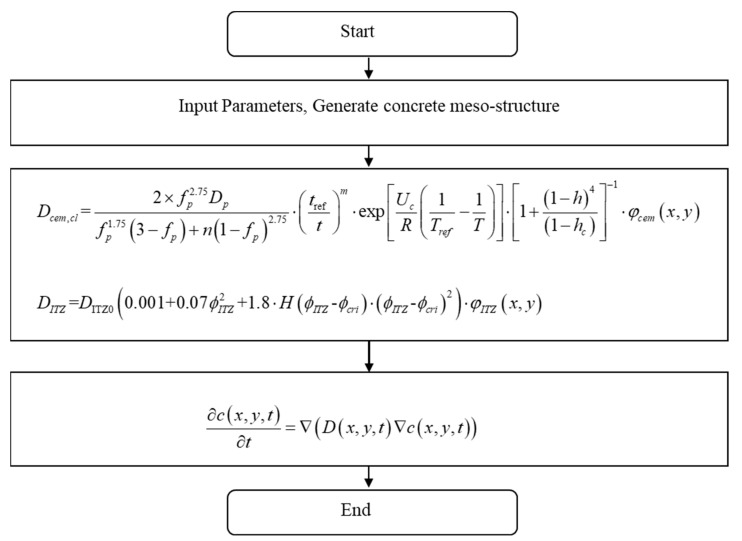
Numerical solution flow chart.

The governing equation of chloride diffusion in concrete is shown in Equation (3). In this paper, the central difference method is used to solve Equation (3). The detailed derivation process of the standardized mathematical formula for this kind of problem is given in the literature [37,40], which would not be repeated here. Figure 3 shows the solution flow chart. Table 1 shows the relevant parameters used in the numerical simulation.

(4) $$\frac{\partial c\left(x,y,t\right)}{\partial t}=\frac{\partial }{\partial x}\left(D\left(x,y,t\right)\frac{\partial c\left(x,y,t\right)}{x}\right)+\frac{\partial }{\partial y}\left(D\left(x,y,t\right)\frac{\partial c\left(x,y,t\right)}{y}\right)$$

### 3.2. Verification of the Proposed Model

**Figure 4:**
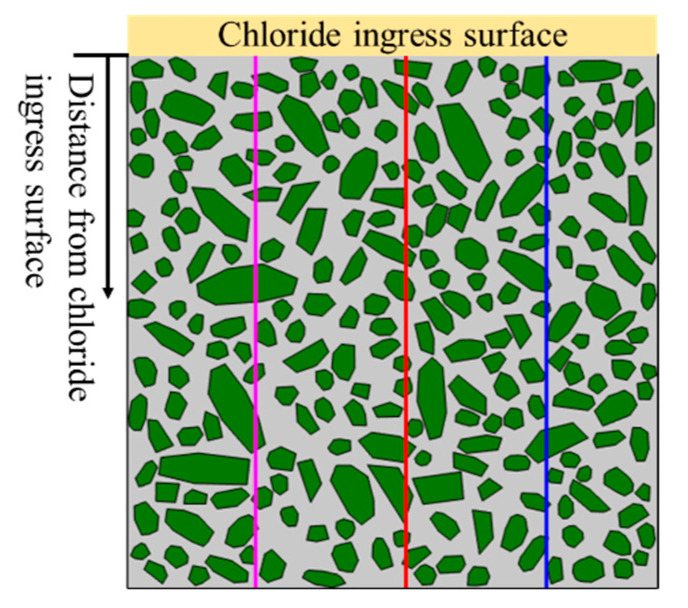
Schematic diagram of distance from chloride ingress surface.

**Figure 5:**
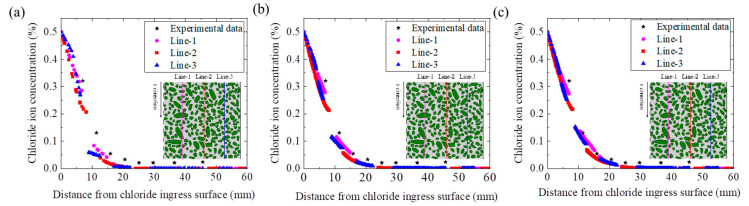
The profile of chloride concentration distribution (**a**) without ITZ, (**b**) the ITZ thickness = 50 μm, (**c**) the ITZ thickness = 100 μm, respectively.

The reliability of the chloride diffusion numerical model proposed in this paper is verified by comparing the experimental data of Fu et al. [41,42] and the numerical simulation results. The values of relevant parameters in the numerical model are listed in Table 2. Moreover, to reduce the influence of randomness of concrete meso-structure, we selected three data lines (e.g., Line-1, Line-2, and Line-3) for comparison, as shown in Figure 4. Figure 5 shows the distribution curve of chloride concentration with depth under the three working conditions (e.g., without ITZ, the ITZ thickness = 50 μm and the ITZ thickness = 100 μm). Obviously, the experimental data are distributed on both sides of the numerical simulation data, and the experimental data are very close to the numerical simulation data. It is indicated that the model proposed in this paper has high reliability. Moreover, the chloride concentration distribution considering the effect of ITZ is in better agreement with the experimental data.

## 4. Simulation Results and Discussion

### 4.1. The Effect of Coarse Aggregate Rate on Chloride Diffusion

**Figure 6:**
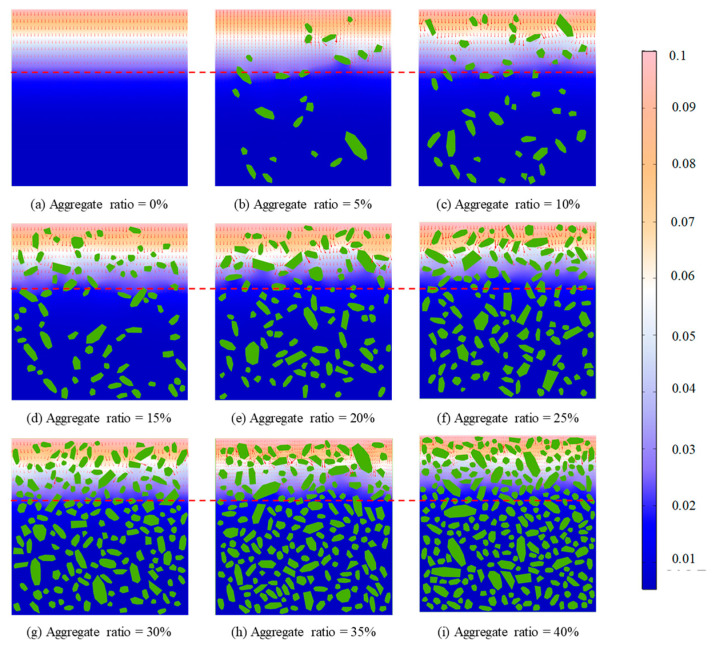
Chloride concentration distribution for different coarse aggregate rate conditions.

**Figure 7:**
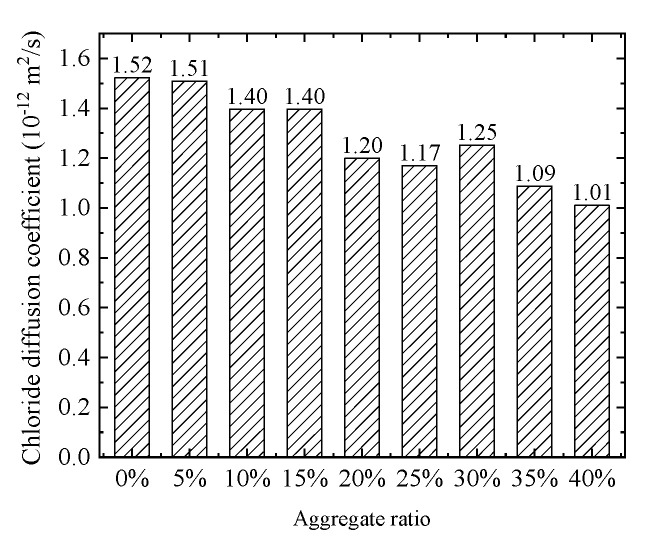
Equivalent chloride ion diffusion coefficient.

**Figure 8:**
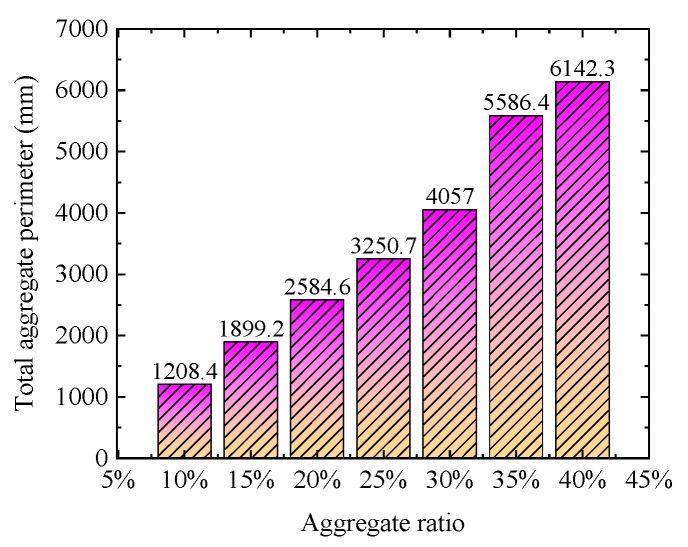
The total perimeter of the coarse aggregate for different aggregate ratios.

The chloride concentration distribution for different coarse aggregate rat is shown in Figure 6. Obviously, the chloride concentration decreases gradually with the increase of the coarse aggregate ratio. Moreover, compared with the chloride diffusion path of pure cement mortar, the chloride diffusion path is more tortuous with the increase of coarse aggregate ratio. This indicates aggregate hinders chloride diffusion. Furthermore, the histogram of the equivalent chloride diffusion coefficient is demonstrated in Figure 7. However, the effective chloride diffusion coefficient with a 30% coarse aggregate ratio is slightly higher than that with a 25% coarse aggregate ratio. This may be caused by the randomness of concrete aggregate, but the general trend is that the effective diffusion coefficient of concrete decreases with the increase of aggregate ratio, which is consistent with the experimental phenomenon of Yang et al. [43]. In addition, the essential reason for the tortuosity of chloride diffusion is that chloride diffusion needs to bypass the coarse aggregate. Therefore, the tortuosity of chloride diffusion can be reflected by calculating the total perimeter of coarse aggregate. The total perimeter of the aggregate for different coarse aggregate ratios is shown in Figure 8. Obviously, with the increase in coarse aggregate ratio, the total perimeter of coarse aggregate increases. Hence, the chloride diffusion path is more tortuous with the increase of coarse aggregate ratio.

**Figure 9:**
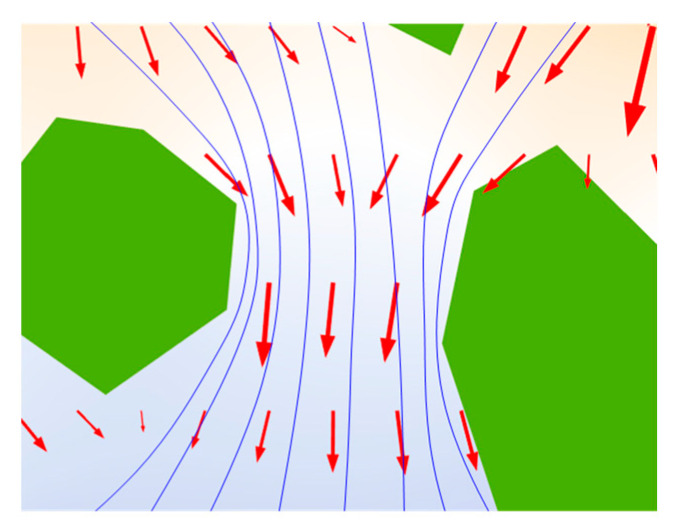
Local chloride ion diffusion.

Moreover, by comparing the chloride diffusion paths, coarse aggregate not only hinders the chloride diffusion, but also has an agglomeration effect on chloride distribution. Figure 9 shows an enlarged view of the local chloride ion diffusion path for 30% of the coarse aggregate. As well, the agglomeration of coarse aggregate increases the chloride ion concentration in the cement mortar.

**Figure 10:**
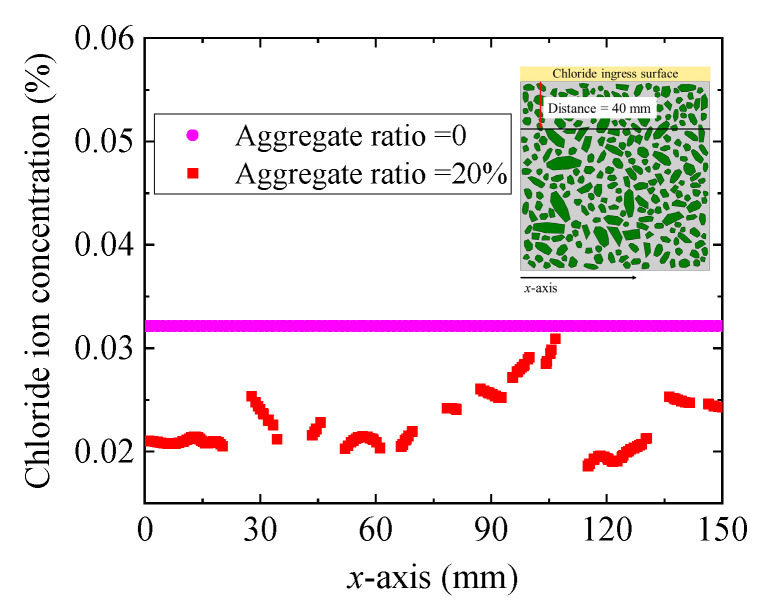
Chloride concentration distribution at the 50th.

Figure 10 shows the distribution of chloride concentration at 40 mm away from the ingress surface. When the concrete is simplified to a single-phase material (e.g., aggregate ratio = 0), the chloride concentration is the same at the same distance from the chloride ingress surface, as demonstrated in Figure 10. However, when the chloride is regarded as a multiphase composite, the chloride concentration has a great difference at the same distance from the ingress surface, as dominated in Figure 10. For example, when the coarse aggregate ratio is 40%, the average chloride concentration at 40 mm from the chloride ingress surface is 0.027% and the standard deviation is 0.0022%. Therefore, when we study the corrosion of reinforcement caused by chloride ingress, it should be considered the randomness of chloride concentration distribution on the surface of reinforcement. It is a reliable method to analyse the durability failure of reinforced concrete caused by chloride ingress by statistical and probability methods.

### 4.2. The Effect of Different ITZ Thickness

**Figure 11:**
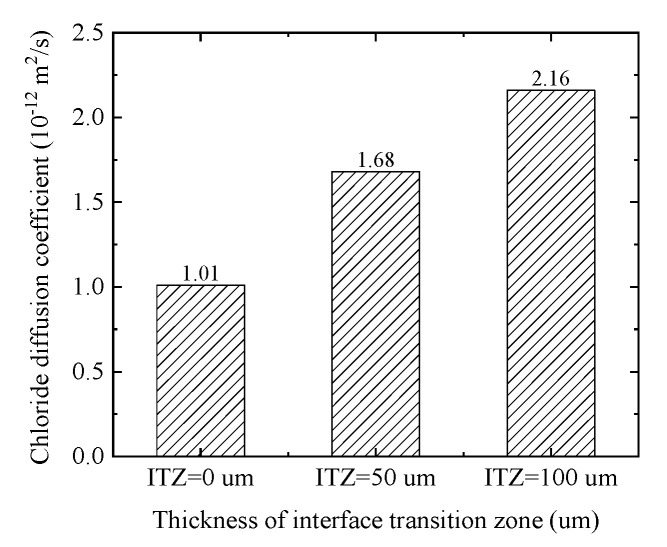
Equivalent chloride diffusion coefficients for different ITZ thicknesses.

The ITZ thickness is related to cement type, construction technology, curing method, and other factors, and its range is between 20 μm and 100 μm. To quantitatively analyze the influence of the ITZ thickness on the chloride diffusion, the diffusion process of chloride in concrete with different thicknesses of ITZ (i.e., 0 μm, 50 μm, 100 μm) is simulated in this paper. From the equivalent chloride diffusion coefficient histogram as shown in Figure 11, With the increase of the ITZ thickness, the equivalent chloride diffusion coefficient is gradually increasing. Furthermore, the equivalent chloride diffusion coefficients with the ITZ thickness of 50 μm and 100 μm are 65% and 104% higher than that without considering the ITZ effect. Moreover, as can be seen from Figure 11, the ITZ thickness has a linear relationship with the effective chloride diffusion coefficient. Moreover, in the above two cases, the total area of ITZ accounts for only 0.135% and 0.278% of the total area of concrete, respectively. It is indicated that although the area of the ITZ can be ignored, the ITZ has a very important influence on chloride diffusion, and this influence increases gradually with the increase of ITZ thickness.

**Figure 12:**
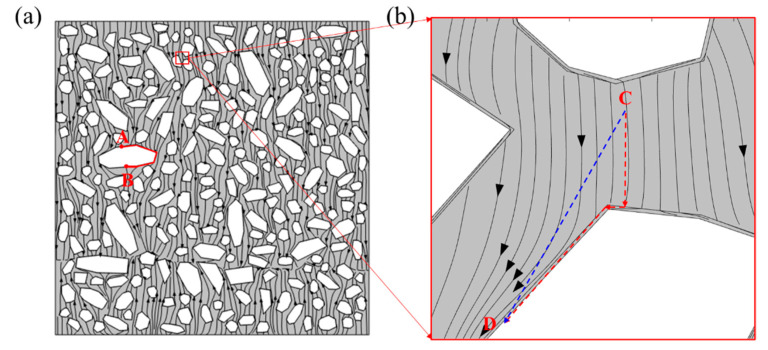
Chloride diffusion trajectories in meso-structures of concrete. (**a**) Overall concrete section (**b**) Local amplification.

More importantly, the diffusion trajectories of chloride in the concrete meso-structures have been clearly presented in Figure 12. Obviously, the aggregate hinders the diffusion of chloride and increases the diffusion path of chloride. For instance, the chloride ions at point A cannot diffuse through the aggregate to point B along a straight line, but must diffuse around aggregate to point B. Moreover, it is found that the ITZ provides a fast channel for chloride diffusion, as demonstrated in Figure 12b. Consequently, the mechanism of ITZ promoting chloride diffusion in concrete can be well understood by this phenomenon.

### 4.3. Chloride Diffusion Characteristics in ITZ

**Figure 13:**
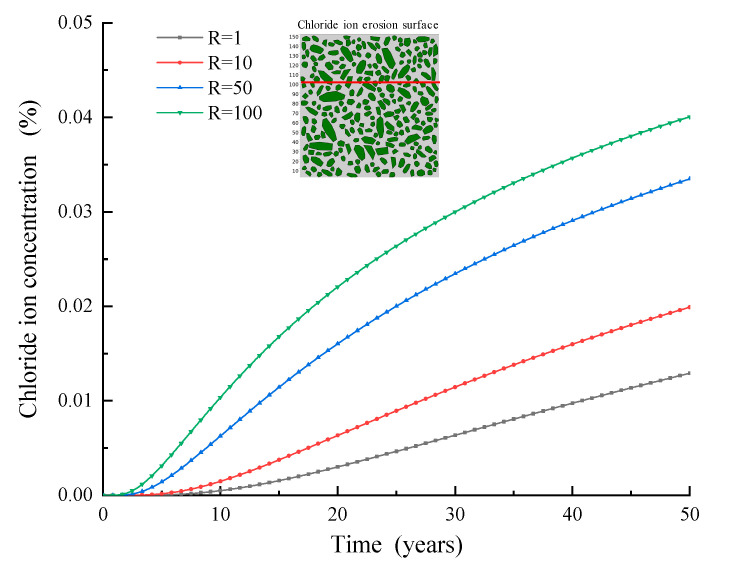
Chloride ion concentration–time curve (R = 1\10\50\100).

It is worth mentioning that the diffusion characteristic of ITZ is another important factor affecting chloride diffusion behavior. The diffusion characteristics of chloride in the ITZ are related to many factors, such as water-cement ratio, construction technology, curing conditions, cement varieties, and so on. In this paper, the ratio (e.g., R) of the chloride diffusion coefficient in the ITZ to that in the cement mortar phase is used as the index to evaluate the chloride diffusion performance in the ITZ. Obviously, the greater the R value is, the better the chloride diffusion performance is. Figure 13 shows the variation curve of chloride concentration on the reinforcement surface under R = 1/10/50/100. Obviously, with the increase in R value, the chloride concentration is also increasing on the reinforcement surface.

**Figure 14:**
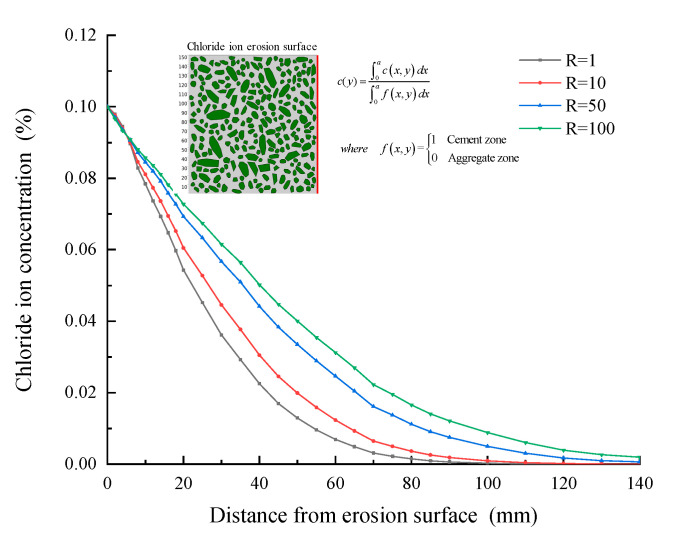
Chloride ion concentration–depth curve (R = 1\10\50\100).

Moreover, the chloride concentration distribution curve along with the ingress depth at the 50th year is shown in Figure 14. The chloride concentration increases with the increase of chloride diffusion coefficient in ITZ. It is worth noting that the chloride concentration distribution curve with R = 50 is not in the middle of the two curves with R = 1 and R = 100. This further shows that the chloride concentration distribution is not directly proportional to the chloride diffusion coefficient in ITZ. With the increase of the multiple of the chloride diffusion coefficient in ITZ, the influence of that in the ITZ on the chloride concentration distribution is weakening. This is mainly because the chloride ion concentration is the result of the joint action of cement mortar and ITZ.

## 5. Conclusions

A multi-phase mesoscopic numerical model is proposed to fully explore the mechanism of diffusion behavior of chloride in concrete for predicting the service life of RC structures. Considering the randomness of the ITZ thickness, the existing random coarse aggregate model is developed. Furthermore, the chloride diffusion coefficient model is constructed by introducing the normal random distribution function to consider the randomness of the chloride diffusion coefficient. Moreover, the reliability of the proposed model is validated by third-party experiments. Finally, the effects of coarse aggregate ratio, ITZ thickness, and diffusion performance of ITZ on the chloride diffusion in concrete are studied in this paper. Based on a systematic study, the following conclusions can be drawn:

(a) The coarse aggregate increases the chloride diffusion path and reduces the effective chloride diffusion coefficient in concrete. With the increase in coarse aggregate ratio, the effective chloride diffusion coefficient shows a downward trend. Moreover, coarse aggregate increases the non-uniformity of chloride distribution in concrete, which increases the uncertainty of reinforcement corrosion caused by chloride attack.
(b) Especially, the mechanism of ITZ promoting the chloride diffusion is revealed by simulating the chloride diffusion trajectories in concrete meso-structure. Although the volume content of the ITZ is very small, less than 0.2% of the total volume of concrete, the effect of the ITZ on the diffusion of chloride ions in concrete cannot be ignored.
(c) The effective diffusion coefficient of chloride is also increasing with the increase of the diffusion performance of ITZ. However, with the improvement of chloride diffusion performance in the ITZ, this increasing trend is gradually weakened, which indicates that the influence of the ITZ diffusion performance on the chloride diffusion behavior is limited.

## Figures and Tables

**Table 1:** Numerical simulation-related parameters.

Parameter Symbol	Unit	Value	Implication	Reference
w/c	-	0.35	water-cement ratio	[7,14]
$\alpha_{c}$	-	0.8	degree of cement hydration	[7,14]
$D_{p}$	$m^{2}/s$	$1.07\times 10^{-10}$	diffusion coefficient of chloride ion in water	[21,34]
$\phi_{ITZ}$	-	0.233	average capillary porosity of ITZ	[7]
$D_{ITZ0}$	$m^{2}/s$	$2.03\times 10^{-9}$	corresponding diffusion coefficient in a bulk water	[7]
m	-	0.2	time index	[15,29]
$t_{ref}$	d	28	reference time	[15,29]

**Table 2:** Verifies the model parameters.

Time (t)	W/C	Aggregate Rate	Interfacial Porosity	Time Index
60 (d)	0.53	0.3	0.4	0.2

## Data Availability

The data used to support the findings of this study are available from the authors upon request.

## References

[1] Liu Q., Hu Z., Wang X., Zhao H., Qian K., Li L., Meng Z. (2022). Numerical Study on Cracking and Its Effect on Chloride Transport in Concrete Subjected to External Load. Constr. Build. Mater..

[2] Zacchei E., Nogueira C.G. (2019). Chloride Diffusion Assessment in RC Structures Considering the Stress-Strain State Effects and Crack Width Influences. Constr. Build. Mater..

[3] Martín-Pérez B., Zibara H., Hooton R., Thomas M.D. (2000). A Study of the Effect of Chloride Binding on Service Life Predictions. Cem. Concr. Res..

[4] Bao J., Wei J., Zhang P., Zhuang Z., Zhao T. (2022). Experimental and Theoretical Investigation of Chloride Ingress into Concrete Exposed to Real Marine Environment. Cem. Concr. Compos..

[5] Xu L., Zhang Y., Zhang S., Fan S., Chang H. (2022). Effect of Carbonation on Chloride Maximum Phenomena of Concrete Subjected to Cyclic Wetting–Drying Conditions: A Numerical and Experimental Study. Materials.

[6] Słomka-Słupik B., Labus K. (2022). Laboratory Test and Geochemical Modeling of Cement Paste Degradation, in Contact with Ammonium Chloride Solution. Materials.

[7] Chen X., Yu A., Liu G., Chen P., Liang Q. (2020). A Multi-Phase Mesoscopic Simulation Model for the Diffusion of Chloride in Concrete under Freeze–Thaw Cycles. Constr. Build. Mater..

[8] Liu Q., Iqbal M.F., Yang J., Lu X., Zhang P., Rauf M. (2021). Prediction of Chloride Diffusivity in Concrete Using Artificial Neural Network: Modelling and Performance Evaluation. Constr. Build. Mater..

[9] Li L., Liu Q., Tang L., Hu Z., Wen Y., Zhang P. (2021). Chloride Penetration in Freeze–Thaw Induced Cracking Concrete: A Numerical Study. Constr. Build. Mater..

[10] Collepardi M., Marcialis A., Turriziani R. (1972). Penetration of Chloride Ions into Cement Pastes and Concretes. J. Am. Ceram. Soc..

[11] Němeček J., Kruis J., Koudelka T., Krejčí T. (2018). Simulation of Chloride Migration in Reinforced Concrete. Appl. Math. Comput..

[12] Yu Z., Chen Y., Liu P., Wang W. (2015). Accelerated Simulation of Chloride Ingress into Concrete under Drying–Wetting Alternation Condition Chloride Environment. Constr. Build. Mater..

[13] Guo B., Li Z., Fu Q., Wang Y., Huang D., Niu D. (2021). Reactive Transport Modelling of Chloride Ingress in Saturated Coral Aggregate Concrete. Front. Mater..

[14] Chen X., Fu F., Wang H., Liang Q., Yu A., Qian K., Chen P. (2021). A Multi-Phase Mesoscopic Simulation Model for the Long-Term Chloride Ingress and Electrochemical Chloride Extraction. Constr. Build. Mater..

[15] Liu Q., Yang J., Xia J., Easterbrook D., Li L., Lu X.-Y. (2015). A Numerical Study on Chloride Migration in Cracked Concrete Using Multi-Component Ionic Transport Models. Comput. Mater. Sci..

[16] Lehner P., Horňáková M., Hrabová K. (2021). Sensitivity Analysis of Stochastic Calculation of SCC Regarding Aggressive Environment. Materials.

[17] Su Z., Li X. (2021). Study on Preparation and Interfacial Transition Zone Microstructure of Red Mud-Yellow Phosphorus Slag-Cement Concrete. Materials.

[18] Bao H., Xu G., Yu M., Wang Q., Li R., Saafi M., Ye J. (2022). Evolution of ITZ and Its Effect on the Carbonation Depth of Concrete under Supercritical CO2 Condition. Cem. Concr. Compos..

[19] Bourdette B., Ringot E., Ollivier J.P. (1995). Modelling of the Transition Zone Porosity. Cem. Concr. Res..

[20] Yang C.C. (2005). Effect of the Percolated Interfacial Transition Zone on the Chloride Migration Coefficient of Cement-Based Materials. Mater. Chem. Phys..

[21] Yang C.C., Cho S.W. (2005). Approximate Migration Coefficient of Percolated Interfacial Transition Zone by Using the Accelerated Chloride Migration Test. Cem. Concr. Res..

[22] Tian Y., Tian Z., Jin N., Jin X., Yu W. (2018). A Multiphase Numerical Simulation of Chloride Ions Diffusion in Concrete Using Electron Microprobe Analysis for Characterizing Properties of ITZ. Constr. Build. Mater..

[23] Wu K., Shi H., Xu L., Ye G., De Schutter G. (2016). Microstructural Characterization of ITZ in Blended Cement Concretes and Its Relation to Transport Properties. Cem. Concr. Res..

[24] Kim H.K., Lee H.K. (2018). Hydration Kinetics of High-Strength Concrete with Untreated Coal Bottom Ash for Internal Curing. Cem. Concr. Compos..

[25] Wu Y., Xiao J. (2018). Multiscale Digital-Image Driven Stochastic Finite Element Modeling of Chloride Diffusion in Recycled Aggregate Concrete. Constr. Build. Mater..

[26] Zheng J., Wong H.S., Buenfeld N.R. (2009). Assessing the Influence of ITZ on the Steady-State Chloride Diffusivity of Concrete Using a Numerical Model. Cem. Concr. Res..

[27] Du X., Jin L., Ma G. (2014). A Meso-Scale Numerical Method for the Simulation of Chloride Diffusivity in Concrete. Finite Elem. Anal. Des..

[28] Zheng B., Li T., Qi H., Gao L., Liu X., Yuan L. (2022). 3D Meso-Scale Simulation of Chloride Ion Transportation in Cracked Concrete Considering Aggregate Morphology. Constr. Build. Mater..

[29] Sun J., Xie J., Zhou Y., Zhou Y. (2022). A 3D Three-Phase Meso-scale Model for Simulation of Chloride Diffusion in Concrete Based on ANSYS. Int. J. Mech. Sci..

[30] Wang Z.M., Kwan A.K.H., Chan H.C. (1999). Mesoscopic Study of Concrete I: Generation of Random Aggregate Structure and Finite Element Mesh. Comput. Struct..

[31] Gao G.Z., Liu Y.D. (2003). Two-Dimensional Random Aggregate Structure for Concrete. J. Tsinghua Univ. (Sci. Technol.).

[32] Liu Q.F., Feng G.L., Xia J., Yang J., Li L.Y. (2018). Ionic Transport Features in Concrete Composites Containing Various Shaped Aggregates: A Numerical Study. Compos. Struct..

[33] Huang Y., Yang Z., Ren W., Liu G., Zhang C. (2015). 3D Meso-Scale Fracture Modelling and Validation of Concrete Based on in-Situ X-Ray Computed Tomography Images Using Damage Plasticity Model. Int. J. Solids Struct..

[34] Jain A., Gencturk B. (2021). Multiphysics and Multiscale Modeling of Coupled Transport of Chloride Ions in Concrete. Materials.

[35] Zhang F., Hu Z., Wei F., Wen X., Li X., Dai L., Liu L. (2021). Study on Concrete Deterioration in Different NaCl-Na2SO4 Solutions and the Mechanism of Cl− Diffusion. Materials.

[36] Jiang W., Shen X., Hong S., Wu Z., Liu Q. (2019). Binding Capacity and Diffusivity of Concrete Subjected to Freeze-Thaw and Chloride Attack: A Numerical Study. Ocean Eng..

[37] Gao S., Guo J., Gong Y., Ban S., Liu A. (2022). Study on the Penetration and Diffusion of Chloride Ions in Interface Transition Zone of Recycled Concrete Prepared by Modified Recycled Coarse Aggregates. Case Stud. Constr. Mater..

[38] Nitka M., Tejchman J. (2020). Meso-Mechanical Modelling of Damage in Concrete Using Discrete Element Method with Porous ITZs of Defined Width around Aggregates. Eng. Fract. Mech..

[39] Shi Y., Lv X., Zhou S., Liu Z., Yang M., Liu C., Lu C. (2021). Mechanical Properties, Durability, and ITZ Characteristics of Full-Grade Dam Concrete Prepared by Aggregates with Surface Rust Stains. Constr. Build. Mater..

[40] Martín-Pérez B., Pantazopoulou S.J., Thomas M.D.A. (2001). Numerical Solution of Mass Transport Equations in Concrete Structures. Comput. Struct..

[41] Ye H., Fu C., Jin N., Jin X. (2018). Performance of Reinforced Concrete Beams Corroded under Sustained Service Loads: A Comparative Study of Two Accelerated Corrosion Techniques. Constr. Build. Mater..

[42] Ye H., Tian Y., Jin N., Jin X., Fu C. (2013). Influence of Cracking on Chloride Diffusivity and Moisture Influential Depth in Concrete Subjected to Simulated Environmental Conditions. Constr. Build. Mater..

[43] Yang C., Su J. (2002). Approximate Migration Coefficient of Interfacial Transition Zone and the Effect of Aggregate Content on the Migration Coefficient of Mortar. Cem. Concr. Res..

